# The Contribution of TSLP Activation to Hyperalgesia in Dorsal Root Ganglia Neurons of a Rat

**DOI:** 10.3390/ijms23042012

**Published:** 2022-02-11

**Authors:** Chun-Ching Lu, Ying-Yi Lu, Hung-Pei Tsai, Chieh-Hsin Wu

**Affiliations:** 1 Department of Orthopaedics and Traumatology, Taipei Veterans General Hospital, Taipei 112, Taiwan; ckluking@gmail.com; 2 Department of Orthopaedics, School of Medicine, National Yang Ming Chiao Tung University, Taipei 112, Taiwan; 3 Department of Dermatology, Kaohsiung Veterans General Hospital, Kaohsiung 813, Taiwan; actinp@hotmail.com; 4 Department of Nursing, Shu-Zen Junior College of Medicine and Management, Kaohsiung 821, Taiwan; 5 Department of Surgery, Division of Neurosurgery, Kaohsiung Medical University Hospital, Kaohsiung 807, Taiwan; carbugino@gmail.com; 6 Department of Surgery, School of Medicine, College of Medicine, Kaohsiung Medical University, Kaohsiung 807, Taiwan

**Keywords:** diameter, nerve fiber, transient receptor potential vanilloid 1 (TRPV1), thymic stromal lymphopoietin (TSLP)

## Abstract

Peripheral nerve injury involves divergent alterations within dorsal root ganglia (DRG) neurons sensitized by persistent inflammation. Thymic stromal lymphopoietin (TSLP) production is crucial in the development of chronic inflammatory responses. Herein, we investigate the changes of TSLP expression in rats’ DRG neurons between injured and uninjured sides in the same rat. Linalyl acetate (LA) was served as a TSLP inhibitor and given intraperitoneally. Rats were assigned to be group of chronic constriction injury (CCI) of the sciatic nerve and the group of CCI of the sciatic nerve administrated with LA. Over 14 days, the rats were measured for paw withdrawal thresholds. DRGs were collected to assess morphological changes via immunofluorescence study. After receiving CCI, the rats rapidly developed mechanical hyperalgesia. TSLP expression at DRG, on the ipsilateral injured side, was consistent with changes in pain behaviors. TSLP appeared in nerve fibers with both small diameters and large diameters. Additionally, TSLP was expressed mostly in transient receptor potential vanilloid-1 (TRPV1)-positive nociceptive neurons. Administration with LA can attenuate the pain behaviors and expression of TSLP in DRG neurons, and in apoptotic neurons at the injured side, but not in the contra-lateral uninjured side. Overall, these results imply that altered expressions of TSLP in nociceptive DRG neurons contributed to mechanical hyperalgesia in a CCI rat model.

## 1. Introduction

Acting as a bridge between somatosensory information and the spinal cord, dorsal root ganglia (DRG) neurons transmit stimuli from peripheral nerve terminals to central nervous structures [1]. DRGs consist of various cells, including macrophages, satellite glial cells and primary sensory neurons [2]. In response to nerve injury, primary sensory neurons adapt their morphology and function to augment the peripheral sensitization and influence expression of nociceptors [3]. Depending on the sizes of cell bodies, DRG neurons are divided into A-neurons (large cell bodies) and C-neurons (small cell bodies). Not only C-neurons (high-threshold Aδ-fibers and C-fibers) but also A neurons (low-threshold Aβ-nociceptors) can relay pain signals [4]. Prolonged inflammation from peripheral nerve injury induces the perception of pain though the activation or sensitization of nociceptors [5]. Neuropathic pain arises from lesions on somatosensory systems and manifests as paresthesia, dysesthesias, allodynia, hyperalgesia, and spontaneous unprovoked pain [6]. It usually occurs simultaneously with sensory loss and weakness [7,8,9] in the same afflicted nerve segment [10], which lacks efficient treatment options [11,12,13]. It compromises quality of life and consumes substantial healthcare resources, leading to major economic consequences [14,15,16,17,18]. Nerve injury induces the release of inflammatory factors at damaged sites, thus further leading to sensory dysfunction within innervated skin. However, the blockade of both A-neurons and C-neurons can prevent mechanical allodynia effectively after nerve injury [19].

Neuro-inflammation, a localized inflammation in nervous systems, leads to enhanced vascular permeability and leukocytes infiltration, thereby activating glial cells and cytokines production [20]. In DRGs, the activation of glial cells and neuronal changes after nerve injury reciprocally promote neuro-inflammation through glial—neuron interaction [21,22,23]. Neuro-inflammation further induces peripheral sensitization and central sensitization, thus resulting in neuropathic pain [24,25]. Thymic stromal lymphopoietin (TSLP) emerges as a cytokine with pleiotropic properties, which is not only involved in various allergic disorders, but is also implicated in chronic inflammatory diseases and cancers [26,27,28,29]. TSLP works through binding to a heteromeric receptor comprising a thymic stromal lymphopoietin receptor (TSLPR) chain and interleukin-7 receptor-α (IL-7Rα) [30,31,32,33]. In a demyelinating disorder of the central nervous system (CNS), TSLP was generated by choroid plexus epithelial cells and spinal cord astrocytes [33]. Through working with the TSLPR expressed on microglia cells, the survival of MHC class II+ immune cells was regulated to influence the myelin-degenerative CNS [34]. By activating sensory neurons directly, and/or targeting sensory neurons indirectly through inflammatory mediators secreted by activated immune cells, TSLP excites itch behaviors [35]. Although pain and itch are distinct sensations, they both relay on a nervous system to transmit and interact. Since nociceptive stimuli relieve itch, pain perception is considered to be dominant over itch [36]. Activation of the nociceptive neural pathways alerts harm signals to induce acute withdrawal behaviors [37].

While TSLP’s action is known to regulate itch sensory neurons, its effect on DRG nociceptive neurons needs further clarification. In this study, we used linalyl acetate (LA) as a TSLP inhibitor to see the changes in TSLP in DRG neurons between the injured and uninjured sides of the same rat in a rat chronic constriction injury (CCI) model.

## 2. Results

### 2.1. Nerve Injury-Induced Expression of TSLP in DRG Neurons

**Figure 1:**
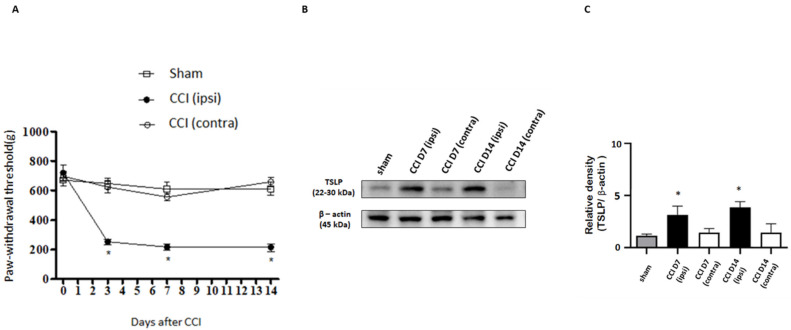
Nerve injury-induced expression of TSLP in ipsilateral DRG neurons. (**A**) The hindpaw-withdrawal threshold (g) was detected using the calibrated forceps test at day 3, 7, and 14 after CCI. The ipsilateral (ipsi) injured sides showed a decreased paw-withdrawal threshold as compared to that in the contralateral (contra) side and sham group after injury. Values are presented as means ± s.e.m. (*n* = 6 rats). * *p* < 0.05; ANOVA test. (**B**) Lumbar 4/5th DRG on both sides were dissected. TSLP proteins were measured by western blot analysis when β-actin was used as the loading control. At day 7 and day 14 after CCI, TSLP proteins were increased in DRG in the ipsilateral injured side as compared to those in the contralateral side and sham group. (**C**) Each band signal density was quantitated and normalized to that of its own β-actin in each side. Values are presented as means ± s.e.m. (*n* = 6 rats). * *p* < 0.05, compared to contra side and sham group, Student’s *t* test.

Sensory stimuli from peripheral tissue are conveyed to the spinal cord by DRG neurons with different sizes depending on their distinct functions [38]. A CCI rat model was established to evaluate nociception via calibrated forceps tests, and sciatic nerve exposure was also performed in a sham group but without sciatic nerve ligation. CCI rats showed mechanical hyperalgesia in the ipsilateral injured side, as compared to the contralateral side in the uninjured and sham group. The paw-withdrawal thresholds between the contralateral side and the sham group were similar (Figure 1A). To assess whether the involvement of TSLP in sensory neurons differed between the ipsilateral injured side and the contralateral side, we examined the changes in TSLP expressed in rat DRGs. Figure 1B shows that 7 days and 14 days after nerve injury, the level of TSLP proteins in lumbar 4/5th DRG from the ipsilateral injured side increased approximately by 3-fold as compared to that from the contralateral uninjured side and the sham group (Figure 1C). These results imply that mechanical hyperalgesia in the ipsilateral injured side of the nerve might be attributed to the changes in TSLP.

### 2.2. Nerve Injury-Induced TSLP Expression in Both Small-Sized and Large-Sized DRG Neurons

**Figure 2:**
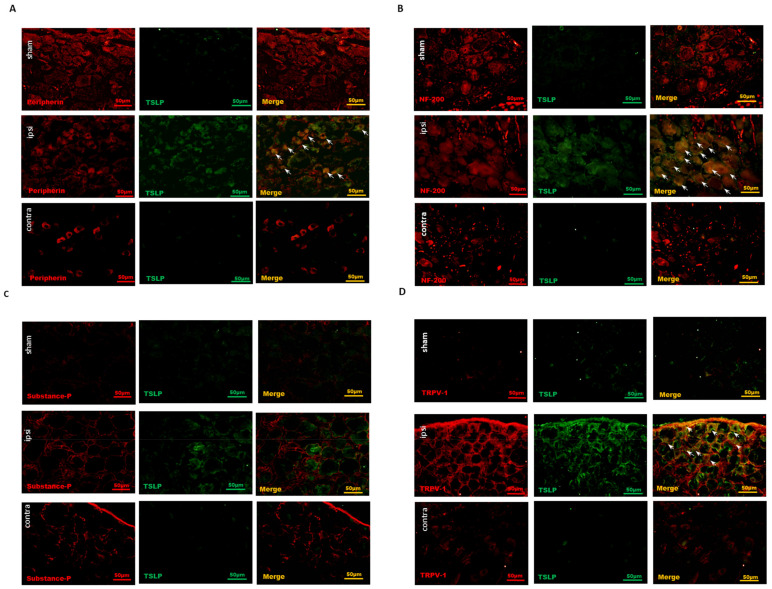
Nerve injury-induced TSLP expression in both small-sized and large-sized DRG neurons, esp. nociceptive DRG neurons. Distribution of TSLP was detected when DRG neurons were double-labeled (yellow) with TSLP (green) and neuronal markers (red). (**A**) Detection of peripherin presented as a small-sized neuronal marker and (**B**) NF200 as a large-sized neuronal marker. TSLP-positive DRG neurons expressed not only peripherin but also NF200 in the ipsilateral (ipsi) injured side as compared to that in the contralateral (contra) side and sham group. (**C**) Detection of substance P presented as a peptidergic non-myelinated neuronal marker and (**D**) TRPV1 as a nociceptive marker. TSLP-positive DRG neurons expressed TRPV1 mostly in the ipsilateral (ipsi) injured side as compared to that in the contralateral (contra) side and sham group. Pairs of merged images are shown on the right panel. White arrows indicate doubled-labeled cells. Scale bars represent 50 μm (*n* = 6 rats).

Most of the time, small-sized neurons are responsible for nociceptive information transmission, although Aβ fibers convey pain stimulus in part [39]. To assess the TSLP expression between large- and small-sized DRG neurons, the prevalence of TSLP-positive DRG neurons co-localized with NF200 (marker for large-sized Aβ fiber neurons) and with peripherin (marker for small-sized Aδ and C-fiber neurons) was examined through immunofluorescence [40,41]. Our results demonstrate that the increased TSLP-positive DRG neurons were not only NF200-positive (Figure 2A) but peripherin-positive (Figure 2B) on the ipsilateral injured side, as compared to that on the contralateral, uninjured side and in the sham group. Overall, the TSLP-positive neurons increased in both small-sized and large-sized DRG neurons from the ipsilateral injured side to convey sensory signals after nerve injury.

### 2.3. Nociceptive DRG Neurons Expressed TSLP after Nerve Injury

Transient receptor potential vanilloid 1 (TRPV1) expressed in DRG sensory neurons exerts as a sensor for endogenous or exogenous noxious stimuli [42]. Functioning as a major nociceptive contributor, the TRPV1 in DRG neurons is activated by inflammatory factors to convey pain signals to the spinal cord [43,44]. TRPV1 is preferentially expressed in small-sized DRG sensory neurons [37]. To examine the cellular subtypes of TSLP-positive neurons, we detected the expressions of various neurons markers, including substance P (SP) for peptidergic DRG neurons and TRPV1 for nociceptive DRG neurons. Most TSLP-positive DRG neurons did not express SP in both sides or in the sham group (Figure 2C). In contrast, TRPV1 was expressed and increased in TSLP-positive DRG neurons from the ipsilateral injured side as compared to that from the contralateral uninjured side and the sham group (Figure 2D). These results indicate that TSLP was upregulated in nociceptive DRG neurons after peripheral nerve injury, contributing to the transmission of pain signals.

### 2.4. LA Decreased Not Only Small-Sized but Also Large-Sized DRG Neurons, Especially Nociceptive DRG Neurons

**Figure 3:**
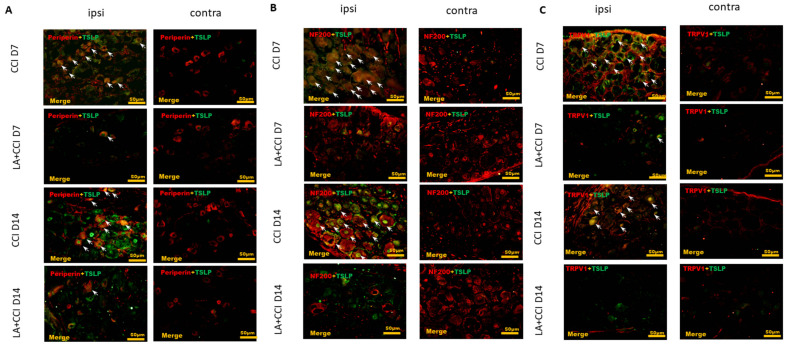
LA decreased not only expression of small-sized but also expression of large-sized DRG neurons, esp. nociceptive DRG neurons. The distribution of TSLP was detected when DRG neurons were double-labeled (yellow) with TSLP (green) and neuronal markers (red). LA serves as a TSLP inhibitor. (**A**) LA decreased the TSLP-positive small-sized DRG neurons, (**B**) TSLP-positive large-sized DRG neurons, and (**C**) TSLP-positive nociceptive DRG neurons in the ipsilateral (ipsi) injured side as compared to that in the contralateral (contra) side. Pairs of merged images are shown in the right panels. White arrows indicate doubled-labeled cells. Scale bars represent 50 μm (*n* = 6 rats).

LA, a main constituent of lavender oil [45], can modulate inflammatory response through downregulating TSLP production, which A23187 and PMA induced in HMC-1 mast cells [46,47,48]. Additionally, LA can ameliorate phorbol myristate acetate (PMA)-induced ear edema by inhibiting TSLP in an allergic mouse model [45,49]. Therefore, to address the function of TSLP in mechanical sensitivity, we administered LA as a TSLP inhibitor at a dose of 100 ug/kg into the rats’ peritoneum before CCI. LA decreased the TSLP-positive small-sized DRG neurons (Figure 3A), TSLP-positive large-sized DRG neurons (Figure 3B) and TSLP-positive nociceptive DRG neurons (Figure 3C) in the ipsilateral side and the injured side, as compared to those in the contralateral uninjured side.

### 2.5. LA Attenuated the Mechanical Hyperalgesia through TSLP/TSLPR Complex

**Figure 4:**
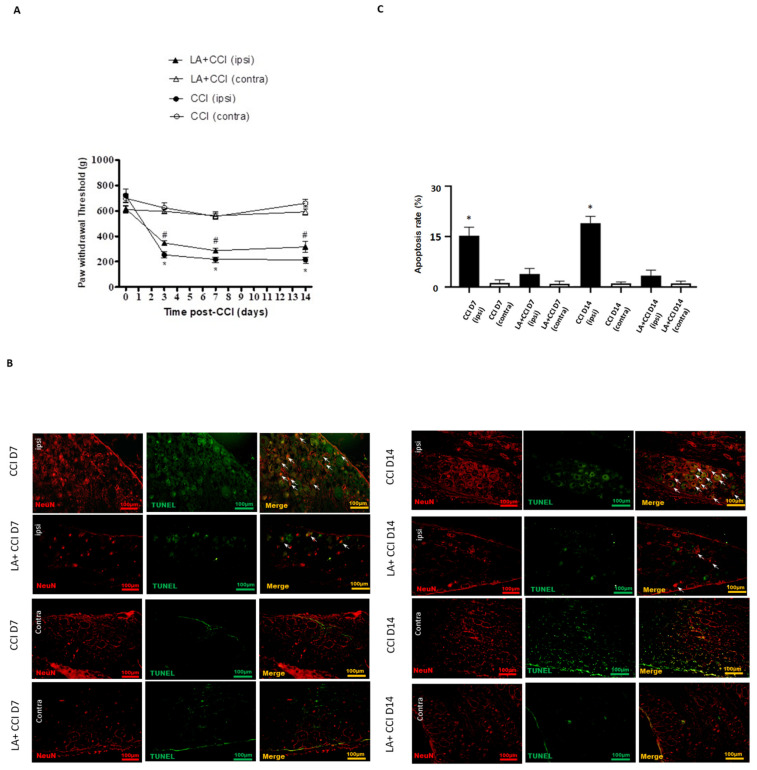
LA attenuated the apoptotic DRG neurons. (**A**) The hindpaw-withdrawal threshold (g) was detected using calibrated forceps test at day 3, 7, and 14 after CCI. LA serves as a TSLP inhibitor. CCI decreased the paw-withdrawal threshold in the ipsilateral (ipsi) injured side as compared to the contralateral (contra) side in rats. Values are presented as means ± s.e.m. (*n* = 6 rats). * *p* < 0.05, compared to CCI group (contra), # *p* < 0.05, compared to CCI group (ipsi); ANOVA test. LA increased the paw-withdrawal threshold in the ipsilateral side. (**B**) Apoptotic neurons were measured when DRG neurons were double-labeled (yellow) with TUNEL (green) and NeuN (red). Pairs of merged images are shown in the right panels. White arrows indicate apoptotic neurons. Scale bars represent 100 μm. (**C**) Merge immunoreactivities were parallel-measured in ipsilateral and contralateral sides of DRG neurons at 7 and 14 days. This expression of apoptotic neurons in the ipsilateral (ipsi) injured side increased after nerve injury and decreased after LA administration as compared to that in the contralateral (contra) side. Values are presented as means ± s.e.m. (*n* = 6 rats). * *p* < 0.05, compared to LA administration group, Students’ *t* test.

Mechanical hyperalgesia was evaluated at 3, 7 and 14 days after administering LA. CCI rats showed mechanical hyperalgesia in the ipsilateral side as compared to the contralateral side. Moreover, LA-injected CCI rats showed an increase in the paw-withdrawal threshold in response to mechanical stimuli in the ipsilateral side, while rats showed no significant change in paw-withdrawal threshold in the contralateral side in response to LA (Figure 4A).

Meanwhile, to determine whether the LA influenced neuronal apoptosis between the DRGs of both sides, which is crucial in nociception transmission, TUNEL staining was also arranged. The results show that apoptotic neurons increased to nearly 15-fold in the ipsilateral injured side on 7 days and 14 days after nerve injury as compared to the contralateral uninjured side. After treating with LA, the apoptotic DRG neurons in the ipsilateral injured side decreased. However, there was no obvious difference in the contralateral side after treating with LA (Figure 4B,C).

**Figure 5:**
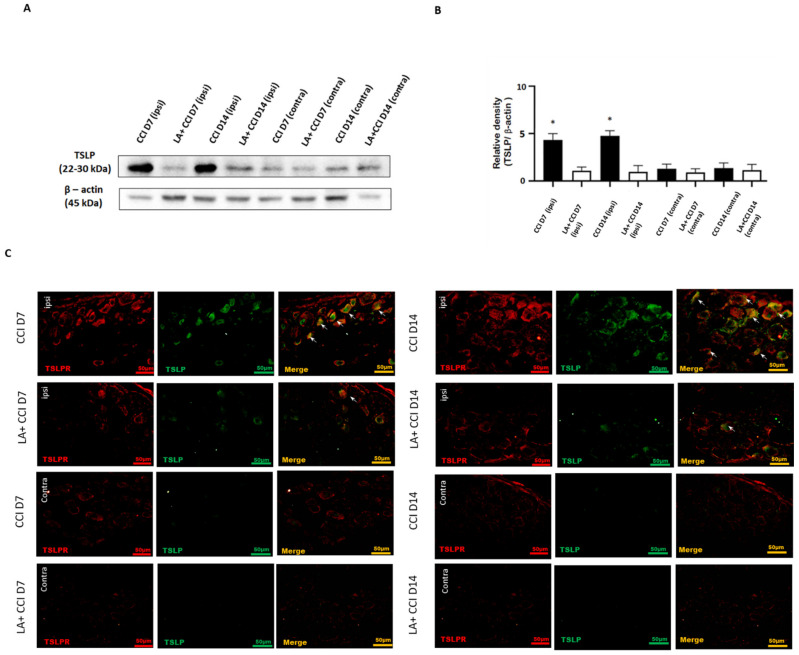
LA attenuated the mechanical hyperalgesia through the TSLP/TSLPR complex. (**A**) Lumbar 4/5th DRG at both sides were dissected. TSLP proteins were measured by western blot analysis when β-actin was used as the loading control. At day 7 and day 14 after CCI, TSLP proteins were increased in DRG in the ipsilateral injured side as compared to those in the contralateral side. LA reduced the TSLP expression in the ipsilateral injured side. (**B**) Each band signal density was quantitated, and normalized to that of its own β-actin in each side. Values are presented as means ± s.e.m. (*n* = 6 rats). * *p* < 0.05, compared to LA administration group, Students’ *t* test. (**C**) The distribution of TSLP was detected when DRG neurons were double-labeled (yellow) with TSLP (green) and TSLPR (red). Merge immunoreactivities were parallel-measured in the ipsilateral and contralateral sides of DRG neurons at 7 and 14 days. This expression of TSLP/TSLPR in the ipsilateral (ipsi) injured side increased after nerve injury, and decreased LA administration as compared to that in the contralateral (contra) side. Pairs of merged images are shown in the right panels. White arrows indicate doubled-labeled cells. Scale bars represent 50 μm (*n* = 6 rats).

Additionally, we examined the changes in TSLP expressed in rat DRGs after the administration of LA (Figure 5A). At 7 days and 14 days after nerve injury, the level of TSLP proteins in the lumbar 4/5th DRG from the ipsilateral injured side increased, and then reduced after LA injection (Figure 5B).

For transmitting nociceptive signals, TSLP requires a distinctive receptor, TSLPR. Therefore, the TSLP and TSLPR expressions in DRG neurons after nerve injury were examined by immunofluorescence staining in parallel studies. The results show that TSLP and TSLPR were co-expressed (Figure 5C). As expected, the expression of TSLP and its receptor TSLPR substantially increased in lumbar 4/5th DRG from the ipsilateral injured side 7 days and 14 days after nerve injury, suggesting that TSLP expression may have functional roles in DRG neurons through working with TSLPR. This increased expression of TSLP/TSLPR in the ipsilateral injured side persisted for at least 14 days after nerve injury. Furthermore, the expression of TSLP/TSLPR in the ipsilateral injured side DRG decreased after administering LA, while rats showed obvious changes in the contralateral side. Taken together, TSLP-positive DRG neurons might convey pain signals through TSLPR; administration with LA could ameliorate mechanical hyperalgesia and apoptotic neurons by decreasing TSLP/TSLPR signals.

## 3. Discussion

The present rat study revealed that mechanical hyperalgesia developed in the ipsilateral side as compared to the contralateral side in rats after nerve injury. The TSLP level in lumbar 4/5th DRGs increased consistently with pain behaviors, too. Notably, TSLP-positive neurons were expressed in nociceptive neurons. Besides this, its expression increased regardless of neuronal size. Administering a TSLP inhibitor attenuated the aforementioned changes. Our findings suggest that the TSLP in nociceptive DRG neurons contributes to mechanical hyperalgesia in the ipsilateral injured side.

Since peripheral nerve injury can induce neurons to release inflammatory mediators to alter neuronal excitability [50,51], the abnormal spontaneous activity of DRG sensory neurons can augment pain sensations in supraspinal areas [52]. Additionally, TSLP is known to work with TSLPR to induce pruritic behaviors by activating sensory neurons with the coordination of inflammatory factors [35]. Studies also indicate that herniated intervertebral disc cells induce the release of pro-inflammatory mediators at damaged intervertebral disc nerves [53], in which process the production of TSLP in discs was correlated with patients’ pain scores. Our results show that the expression of TSLP increased in lumbar 4/5th DRG in the ipsilateral injured side after CCI as compared to the contralateral uninjured site, which was consistent with the mechanical threshold. Neuro-glial interactions further enhance the development of persistent pain [54,55], which initiates glial cell activation or proliferation, or DRG neuron death [56,57,58]. TRPV1 in neurons is critical in regulating neuro-inflammation and transmitting pain signals [59]. Through inflammation mediators, the activation of TRPV1 induces nociceptor sensitization and maintains the sensory neurons’ hypersensitivity, thereby contributing to hyperalgesia. Our study has showed that TSLP increased in TPRV1-positive DRG neurons in the ipsilateral injured side after CCI, and LA decreased TSLP’s involvement in nociceptive sensory neurons, suggesting TSLP-positive nociceptive neurons contribute to mechanical hyperalgesia in CCI rats.

Sensory DRG neurons are classified as large myelinated Aβ, thinly myelinated Aδ and small unmyelinated C fiber neurons [60,61,62]. Mechanical allodynia includes dynamic allodynia, punctate allodynia, and static allodynia, which are transmitted by Aβ, Aδ and C primary sensory fibers, respectively [62,63]. These nociceptors activate in response to noxious stimuli [64]. The A-fibers respond to pain with a short withdrawal latency as compared to slow-conducting C-fibers with a long latency movement. Therefore, A-fibers are responsible for evoking the initial pain response, or sharp pain, whereas C-fibers mediate secondary, or burning, pain [65]. Additionally, A-fibers are important for controlling pain characteristics because they provide the CNS with more information about pain intensity compared to C-fibers [66]. Since TSLP is localized in both large-sized and small-sized DRG neurons, we hypothesize that both sizes of DRG neurons produce the TSLP protein in response to nerve injury to convey pain signals.

In post-herpetic mice, dynamic allodynia has a stronger association with damage in C-fiber neurons compared to damage in A-fiber neurons [67]. However, CCI rats demonstrated that hyperalgesia onset is associated with a preferential loss of large fibers to small myelinated or unmyelinated axons [68]. Axon ligation can cause unmyelinated fiber loss as high as 60% and myelinated fiber loss as high as 80% [14,69]. According to the immunofluorescence staining results in our study, DRG was confirmed by TSLP increases observed in NF200-positive and peripherin-positive neuron cells after CCI, which demonstrates that TSLP was expressed not only in Aβ fiber neurons with thick myelin sheaths, but also in Aδ fiber neurons with thin myelin sheaths and in C-fiber neurons without myelin sheaths. Notably, LA decreased the TSLP involvement in both neuronal sizes, which suggests that neurons in A- and C-fibers both play a key role in pain signaling in accordance with the decreased pain withdrawal threshold after CCI [61,62]. The TSLP in DRG neurons might be able to initiate the pain signal and transmit to the CNS so as to consolidate the pain intensity, which contributes to mechanical allodynia.

After nerve injury, DRG macrophages can release mediators to influence both A-neurons and C-neurons to initiate and maintain mechanical hyperalgesia [70,71]. By the release of inflammatory mediators, DRG sensory neurons also augment pain sensation in supraspinal areas by alterations in neuronal excitability [51,52]. The interaction between neurons and glial cells enhances persistent pain by regulating DRG neuron death [55,56]. Through binding with TSLPR, TSLP can regulate inflammatory signals. Our study shows that TSLP co-expressed with TSLPR in DRG, which increased in the ipsilateral injured side as compared to that in the uninjured side, which is consistent with the changes in apoptotic neurons. LA decreased the apoptotic neurons and the TSLP/TSLPR complex in the ipsilateral injured side. These results imply that TSLP might contribute to neuron death after nerve injury by regulating the TSLPR complex.

However, the pharmacokinetics of LA in rats via intraperitoneal administration remain unclear. Although the current results show that LA improved the mechanical hyperalgesia in CCI rats for 14 days, the relevance of precise rat serum or tissue levels of LA or its metabolites to anti-nociceptive effects is unknown, and needs further study.

## 4. Materials and Methods

### 4.1. Antibodies

The following primary antibodies were used for immunofluorescence assay and western blot analysis: Anti-NF200 was from Sigma-Aldrich (1:50, N5389; Saint Louis, MO, USA), anti-peripherin was from Sigma-Aldrich (1:200, P5117; Saint Louis, MO, USA), anti-TRPV1 was from proteintech (1:500, 66983-1-Ig; Rosemont, IL, USA), anti-TSLP was from Sigma-Aldrich (immunofluorescence: 1:100 and western blot: 1:200, PRS4025; Saint Louis, MO, USA), anti-TSLPR was from Sigma-Aldrich (1:500, WH0064109M3; Saint Louis, MO, USA), anti-substance P was from Abcam (1:500, ab14184; Cambridge, MA, USA), anti-NeuN was from Millipore (1:400, MAB377; Burlington, MA, USA), and anti-β-actin was from Millipore (1:10,000, MAB1501R; Burlington, MA, USA). The following secondary antibodies were used: anti-rabbit IgG HRP (immunofluorescence: 1:500, 111-545-144; Jackson ImmunoResearch, West Grove, PA, USA; western blot: 1:2000, 111-035-444; Jackson ImmunoResearch, West Grove, PA, USA) and goat anti-mouse IgG HRP (immunofluorescence: 1:500, 115-585-146; Jackson ImmunoResearch, West Grove, PA, USA; western blot: 1:2000, AP124P; Jackson ImmunoResearch, West Grove, PA, USA). TUNEL staining was performed by in situ cell death detection kit (11-684-795-910; Roche, NJ, USA).

### 4.2. Experimental Animals

The Institutional Animal Care and Use Committee (IACUC) of the Kaohsiung Medical University approved the animal study (108296). Male Sprague-Dawley rats (8–10 weeks old, body weight: 300–350 g) were purchased from the BioLASCO (Taipei, Taiwan). All animal experimental protocols were carried out in accordance with ARRIVE guidelines and the Kaohsiung Medical University IACUC guidelines. Rats were housed under controlled room temperature and humidity, in a 12-h light–dark cycle with food and water given ad libitum.

To study neuropathic pain after nerve injury, Bennett and Xie’s (1988) unilateral sciatic nerve CCI rat model was generated [72,73]. A total of 60 rats were used for studies and euthanized at the indicated time. The rats were anesthetized intraperitoneally with Zolitil 50 (Virbac; France; 06516), and the right-side sciatic nerve was exposed at the mid-thigh level proximal to the sciatic trifurcation. Without arresting the epineural blood supply, loose ligatures of 4-0 chromic gut (ETHICON; Raritan, NJ, USA; VE601) were tied around the nerve at 1 mm intervals. LA was diluted into PBS and injected into the peritoneum of a rat at the dose of 100 ug/kg before CCI. Rats were assigned as CCI or CCI administrated with LA. The CCI rat group was run in parallel with CCI rats administrated with LA group.

### 4.3. Behavior Responses

Mechanical pain threshold (paw-withdrawal response) was scored using an algometer with calibrated forceps (Bioseb In Vivo Research Instruments, Vitrolles, France). As described by Luis-Delgado et al., the influence of stimulation on each hindpaw was recorded three times [74]. As demonstrated at the time of withdrawal, the maximum force applied to the paw was recorded as the grams (g) of force on the dynamometer. The pain threshold was expressed as the mean ± standard error of the mean (s.e.m.) in each group.

### 4.4. Western Blot Analysis

Lumbar 4/5th DRGs samples were homogenized in lysis buffer (T-PER Tissue Protein Extraction Reagent, Thermo; USA; 78510). The protein contents of supernatant were measured by BCA Protein Assay Kit (Sigma; St. Louis, MO, USA; B9643). Equal amounts of proteins were separated on 8 or 12% SDS-polyacrylamide gel and then transferred to the polyvinylidene difluoride (PVDF) membranes in a transfer buffer after electrophoresis. After the PVDF membranes were blocked at room temperature for 1 h with 5% non-fat milk in Tris-buffered saline Tween-20 (TBST), they were incubated with primary antibody overnight at 4 °C. Then, the membranes were washed with TBST several times, before incubating with the corresponding secondary antibody for 1.5 h at room temperature. Finally, the protein bands were measured by chemiluminescence using an ECL Western Blotting Detection kit and visualized using a MiniChemi™ imaging and analysis system (Beijing, China).

### 4.5. Immunofluorescence Assay

Serial sections of lumbar 4/5th DRGs were dissected and post-fixed in 4% paraformaldehyde overnight, and then transferred into 10, 20 or 30% sucrose in PBS. After washing with PBS several times, the sections were incubated with a blocking buffer containing 1% normal goat serum and 0.1% Triton X-100 in PBS at room temperature. Next, sections were incubated with primary antibodies at 4 °C overnight. On the second day, these sections were washed with PBS solution three times and then incubated with the desired secondary antibodies at room temperature for 2 h. Images were obtained using a fluorescence microscope (Olympus, State College, PA, USA, U-RFL-T). Fluorescence intensity was scored using the Image J software (National Institutes of Health, Bethesda, MD, USA).

### 4.6. Data Analyses

All analyses were undertaken by experienced trained technicians blinded to the study groups. Data expressed as mean + s.e.m. were calculated and analyzed using SPSS (V24.0) statistical software (SPSS, Inc., Chicago, IL, USA) in the study. Comparisons between two groups were performed by Student’s *t* test, and those among multiple groups were performed using two-way ANOVA following normal distribution. Values of *p* less than 0.05 were indicated as statistically significant. The representative values were derived from at least three tests.

## 5. Conclusions

Our data show that TSLP-positive nociceptive DRG neurons increased in the ipsilateral side after nerve injury. Additionally, TSLP increased in thickly myelinated Aβ neurons, thinly myelinated Aδ neurons and in unmyelinated C-fiber neurons, which are the primary afferent neurons involved in the transmission of noxious stimuli. LA treatment ameliorated these changes. That is, the expression of TSLP in primary sensory neurons showed a strong relevance to mechanical hyperalgesia, which offers evidence that we can regulate DRG neurons with varying diameters in pain signaling through regulating the TSLP/TSLPR complex.

## Data Availability

All data generated or analyzed during this study are included in this published article.

## Abbreviations

CNS	central nervous system
CCI	chronic constriction injury
contra	contralateral
DRG	dorsal root ganglia
IL-7Rα	interleukin-7 receptor-α
ipsi	ipsilateral
LA	linalyl acetate
PMA	phorbol myristate acetate
SP	substance P
TSLP	thymic stromal lymphopoietin
TSLPR	thymic stromal lymphopoietin receptor
TRPV1	transient receptor potential vanilloid-1
TBST	Tris-buffered saline Tween-20
